# 

**Published:** 1817-05

**Authors:** 


### CATALOGUE OF MEDICAL BOOKS.

An Essay on Burns, in Two Parts, principally on those which happen to Workmen in Mines from the Explosions of Carburetted Hydrogen Gas; containing, also, a View of the Opinions of Ancient and Modern Authors upon the Treatment of Accidents by Fire, and including a Variety of Cases conducted upon different Principles; the whole tending to secure the Healing Art from Empiricism, and to reduce it to Established Laws. The First Part originally published in 1797; the Second Part in 1800. By Edward Kentish, M.D. Physician to the Bristol Dispensary and St. Peter's Hospital.

A Physiological System of Nosology, with a corrected simplified Nomenclature. By John Mason Good, F.R.S. &c.

---

### *TO SUBSCRIBERS.*

*The increased value of the Journal, by the prospect of a General Index, having caused many applications for the back numbers, the Proprietors feel bound in gratitude to pay some consideration to the accumulated expences thus entailed on many of their earliest subscribers in completing their sets; some Gentlemen, by absence in the service of their country, have been unable to procure the work during certain periods, and others, not aware of the increasing value of their early numbers, have suffered them to be mislaid or destroyed. Any of a date previous to* 1817 *will, therefore, be sold at the reduced price of* 1*s.* 6*d. each, until the* 1*st of October next; after which, none can be had under* 2*s.* 6*d. Some of the numbers are nearly out of print; in supplying these, a preference will of course be given to the earliest application.*

---

### *NOTICES TO CORRESPONDENTS.*

*Several favours from Correspondents have been received, and also several books for our Analysis. The first will appear in our next Number; the latter will be noticed in the order in which we have received them.*

*Our Correspondents will please to be particular in the Address of their Communications, which should be thus,* " To the Editors of the London Medical and Physical Journal, at Mr. Souter's, No. 1, Paternoster-Row; or at Mr. Adlard's, 23, Bartholomew-Close."

